# Bilateral Wilms tumour: a review of clinical and molecular features

**DOI:** 10.1017/erm.2017.8

**Published:** 2017

**Authors:** Jocelyn Charlton, Sabine Irtan, Christophe Bergeron, Kathy Pritchard-Jones

**Affiliations:** 1 UCL Institute of Child Health, University College London, London, UK; 2 Paediatric Surgery Department, Trousseau Hospital, Paris, France; 3 Centre Léon Bérard, Institut d'Hématologie et d'Oncologie Pédiatrie, Lyon, France

## Abstract

Wilms tumour (WT) is the most common paediatric kidney cancer and affects approximately one in 10 000 children. The tumour is associated with undifferentiated embryonic lesions called nephrogenic rests (NRs) or, when diffuse, nephroblastomatosis. WT or NRs can occur in both kidneys, termed bilateral disease, found in only 5–8% of cases. Management of bilateral WT presents a major clinical challenge in terms of maximising survival, preserving renal function and understanding underlying genetic risk. In this review, we compile clinical data from 545 published cases of bilateral WT and discuss recent progress in understanding the molecular basis of bilateral WT and its associated precursor NRs in the context of the latest radiological, surgical and epidemiological features.

## Introduction

Wilms tumour (WT) is a rare kidney cancer that occurs almost exclusively in childhood, with a prevalence of one in 10 000 children younger than 15 years of age. This embryonal tumour generally shows mimicry of cell types seen during normal nephrogenesis, with the classical ‘triphasic’ WT comprising undifferentiated blastemal cells with differentiation towards both stromal and epithelial elements. The genetics of the embryonal tumours of childhood underpinned Knudson's two-hit hypothesis for cancer generation whereby a tumour suppressor gene is silenced by either germline or random somatic loss-of-function mutation of one allele, with the remaining allele lost as a second event post-natally. Hereditary cases are predicted to occur earlier and be more likely to present bilaterally in paired organs such as the kidney. However, when the first WT gene (*WT1*) was identified, it was found to account for only a minority of bilateral and familial WT cases. Indeed, genetic predisposition to WT is uncommon (~5% of all cases) and can be owing to one of several different genetic or epigenetic changes (Ref. 1, 2). With the recent discovery of many new WT genes, the proportion with known genetic predisposition may increase, especially if some have low penetrance (Refs 3, 4, 5).

WTs presenting as bilateral disease can be associated with early disruption in renal development, not only because of involvement of both kidneys but due to the fact that in nearly all cases, tumours are associated with the presence of precursor lesions termed nephrogenic rests (NRs). NRs are clusters of residual embryonic renal cells persisting in a mature kidney that result from incomplete differentiation of metanephric blastema into mature renal parenchyma (Refs 6, 7). Two types of NR are recognised based on morphological features and anatomical location within the kidney. Intralobar NR (ILNR) are usually observed singularly and show predominant stromal composition and often mature fat cells with irregular, indistinct borders and are located towards the renal medulla whereas perilobar NR (PLNR) are often numerous and diffuse located towards the periphery of the renal lobule composed predominantly of blastemal cells with well-defined borders that develop epithelial structures and sclerosis with age (Refs 6, 7). Nephroblastomatosis is defined as the presence of multiple or diffuse NR. In unilateral WTs, NRs are usually only detectable by histology whereas in bilateral WT, the proliferating NRs may be large enough to be seen on imaging (Ref. 8). The term ‘bilateral disease’ is used to encompass bilateral WT, WT in one kidney with nephroblastomatosis in the other, or bilateral nephroblastomatosis, as these cannot always be easily distinguished on imaging. Whilst NRs are considered benign and can regress spontaneously or under chemotherapy, they have a significant risk of progression to WT (Ref. 9).

Bilateral disease can be synchronous (both kidneys affected at the same time) or metachronous (one affected after the other), which occurs in 6.3 and 0.85% WT patients respectively (Ref. 10) with an overall frequency of ~5 to 8% (Refs 11, 12). In general, PLNRs are associated with synchronous bilateral WT, whereas ILNRs are more strongly associated with metachronous WT (Ref. 6). As expected from Knudson's two-hit model, the median age of onset of bilateral WT is younger than for unilateral WT – under 2 years compared with 38 months. What remains unexplained is the remarkable female excess seen in bilateral WT (Ref. 12). Furthermore, the bimodal distribution of age at onset implies a genetic complexity that is as yet only partially understood. For both unilateral and bilateral WT, age at diagnosis is affected by the presence of NRs, patient sex (males are diagnosed on average 6 months earlier than females), underlying syndromes and laterality (Refs 6, 10).

At present, bilateral disease is treated with pre-operative chemotherapy at time of diagnosis followed by surgery. A major clinical challenge is to decide the best time for nephron-sparing surgery (NSS) and if and when there may be value in intensifying or prolonging pre-operative chemotherapy. Thus far, response assessment is based purely on tumour shrinkage. However, it is recognised that the stromal subtype of WT, common in children with *WT1* mutant tumours, may not shrink and may even show a paradoxical increase in tumour size owing to rhabdomyoblastic differentiation, even though it is a favourable histological subtype. Hence, having a technique that could monitor histological response during pre-operative chemotherapy would be useful in planning NSS. Advanced functional imaging using apparent diffusion coefficient (ADC) is a new approach that has the potential to make this distinction (Ref. 13). Furthermore, while WT needs to be surgically removed (Ref. 14), NRs may be left within a patient in some circumstances making their distinction from WTs essential for effective treatment. Patients with bilateral disease need to maintain maximal renal function to ensure longevity requiring advanced imaging and surgical techniques. Here, we review the most recent advances in these fields and explore the molecular biology aspects of bilateral WT.

## Search strategy and selection criteria

References for this Review were identified through searches of PubMed, using appropriate search terms for each section, for the period from 1990 until August 2016 (‘Nephroblastoma’ or ‘Wilms’, ‘Bilateral’ and ‘Nephroblastomatosis’). For the surgical section, only reviews by national or cooperative groups were included because of a recent comprehensive review of this aspect published in 2009 (Ref. 15). Only papers published in English were reviewed. The final reference list was generated on the basis of originality and relevance to the broad scope of this Review.

## WT predisposition syndromes

Unlike adult carcinomas where cells have a lifetime to accumulate damage, the embryonic tumours of childhood are felt to represent random spontaneous genetic changes in a pool of cells that retain the pluripotent differentiation potential of their embryonic counterparts. However, in certain cases, a germline mutation predisposes to WT onset by either providing the first tumour suppressor gene ‘hit’, as previously discussed, or by causing sustained proliferation of renal precursors providing an optimal environment for a second transforming event. Not surprisingly, a much higher frequency of bilateral disease is observed in patients with predisposition syndromes.

Approximately 5% of WTs are associated with known constitutional predisposition syndromes; whilst over 100 syndromic associations are described (Ref. 1), the commoner ones fall into two major categories: those associated with genito-urinary malformation because of underlying abnormalities in the *WT1* gene (WT with Aniridia, Genitourinary abnormalities and mental Retardation (WAGR) syndrome; Denys–Drash syndrome (DDS)) and those associated with an overgrowth phenotype [Beckwith–Wiedemann syndrome (BWS) and Perlman syndrome].

WAGR syndrome is associated with 11p13 deletion encompassing the *WT1* gene. The size of the deletion varies, with mental retardation observed in patients with large deletions. Subsequent to germline *WT1* loss, the second somatic event leading to WT formation in patients with WAGR syndrome is commonly intragenic *WT1* mutation, rather than a second 11p genomic loss, as the latter is likely to be cell lethal. Of children born with WAGR syndrome, 45–57% develop WT (Refs 16, 17). A range of germline intragenic *WT1* mutations have been associated with DDS with the majority affecting the *WT1* DNA-binding domain, specifically within exon 9 (Ref. 18). Although the penetrance of WT in children with constitutional *WT1* mutation is likely much lower, around 74% children with the classical DDS triad develop WT, often with associated ILNRs (Ref. 18) (using the original narrow phenotypic definition of DDS and not including the more recently broadened phenotype with milder renal dysfunction/genitourinary abnormalities with *WT1* mutation).

Germline aberration of *WT1* is clearly associated with increase in bilateral disease as the overall rate of bilateral WT is 5% whereas patients with DDS show incidence of 20% (Ref. 18), and WAGR 17% (Ref. 19). A similarly high frequency (17.3%) of bilateral disease is observed in patients with BWS (Ref. 20), who show germline loss of imprinting (LOI) at 11p15, affecting *IGF2* and *H19* either by gain of DNA methylation or uniparental isodisomy giving two copies of the active paternal *IGF2* allele. However, penetrance is much lower for BWS patients, as only 7.5% develop WT (Ref. 20), often associated with PLNRs. Perlman syndrome is similar to BWS in that both syndromes cause overgrowth, however, the greatest overall frequency of bilateral disease is observed in association with Perlman syndrome. Of the children who survive the prenatal period, 64% develop WT and 55% of these are bilateral. Perlman syndrome is associated with *DIS3L2* mutation and frequently observed with nephroblastomatosis. Other germline genetic anomalies have been associated with bilateral WT, including duplication of 2p24.3 encompassing genes *DDX1* and *MYCN* (Ref. 21), *de novo* t(5;6)(q21;q21) affecting *HACE1* (Ref. 22) and mosaic variegated aneuploidy (Refs 23, 24).

None of these predisposing syndromes show 100% association with bilateral disease, as there is a requirement for a second event prior to tumour formation. The frequency of bilateral disease may be associated with the developmental timing at which the primary aberration occurs. For patients with Perlman syndrome, a very high number develop WT in one or both kidneys whereas for BWS this is much lower suggesting that, on a background of germline *DIS3L2* mutation, a transforming second event occurs more readily, whereas on a background of IGF2 overexpression and H19 loss, there is less selection pressure for transformation. Another potential confounder is the presence of mosaicism in patients, where certain tissues may carry the aberration and others not, and even certain cells within the tissue, if the aberration occurs late in development.

### Molecular features of bilateral WT (Fig. 1)

#### *WT1* and bilateral WT

*WT1* mutation is observed in ~12% sporadic WTs (Ref. 25) and germline *WT1* mutation or loss significantly increases the likelihood of developing bilateral disease. In a comprehensive review of 117 published WT cases with germline WT1 alterations, the authors showed a frequency of bilateral WT in 24, 17 and 52% of the deletion, missense and truncation mutations groups (Ref. 26). When the truncation group was subdivided further, the frequency of bilateral WT was 50% for patients with frameshift and 54% for patients with nonsense mutations (Ref. 26). Two studies that performed *WT1* analysis in large cohorts of nonsyndromic patients with WT found that 8/201 (4%) (Ref. 27) and 6/282 (2%) (Ref. 28) patients had constitutional *WT1* mutation with three and two of these having bilateral disease, respectively. This shows that a relatively low frequency of cases thought to be sporadic may in fact be germline, despite the patients showing no other obvious clinical phenotype.

**Figure 1.**
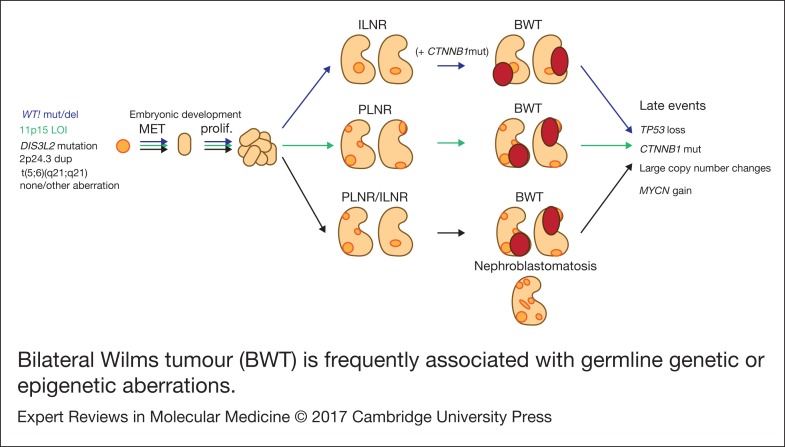
Bilateral Wilms tumour (BWT) is frequently associated with germline genetic or epigenetic aberrations. During kidney development, kidney precursor cells undergo mesenchymal to epithelial transition (MET) to form the epithelial structures of the normal kidney (light orange). In cases where cells carry germline aberrations (but not in every case), normal development is disrupted and retained embryonic tissue is found in the normal kidney (nephrogenic rests; dark orange). Intralobar nephrogenic rests (ILNR) are associated with WT1 mutation and perilobar nephrogenic rests (PLNR) are associated with 11p15 loss of imprinting (LOI). These lesions are considered precursors to Wilms tumour and are found in nearly all cases of bilateral Wilms tumour (BWT; dark red) although the molecular mechanisms involved in transformation are unknown. Mutation of CTNNB1 is likely to be a secondary event following germline WT1 mutation. Further late events are acquired over the progression of the tumour. Shown in black are several reported germline aberrations found in patients with BWT, however the genetic background is not always known and BWT could also arise from somatic mutation in each kidney.

Taking the opposite approach, another study focused specifically on assessment of germline *WT1* status in patients with bilateral disease. By targeted sequencing of *WT1* in eight bilateral WTs (defined in this case as only synchronous bilateral tumours), three patients were found to have germline heterozygous nonsense mutations in *WT1* exon 8, leading to *WT1* protein truncation with no wild-type allele present in the tumours (Ref. 29). The other five patients had no *WT1* mutation and were not further characterised for germline or somatic mutation of other WT genes. A separate study described a much higher frequency, with seven of eight patients with bilateral disease (defined here as either WT in each kidney or WT with NR in the other kidney) showing germline *WT1* mutation (Ref. 30). The final patient had BWS and no *WT1* mutation (Ref. 30). Of the seven *WT1* germline mutant cases, three patients relapsed; all of whom initially had WT and one NR in the contralateral kidney. Two patients developed WT in the kidney with previous NR and one patient developed bilateral WTs. As one of these recurrences was 11 years later, the authors suggest careful follow up for patients with bilateral disease. Although no molecular analysis was performed on the recurrences, the authors did look for *CTNNB1* mutation in the tumours and NRs. It has been hypothesised that *WT1* mutation is an initiating event and *CTNNB1* mutation a secondary event in WT tumourigenesis as *WT1* mutations have been identified in both NRs and WTs, but *CTNNB1* mutations only in the associated WTs (Ref. 31). However, the data shown in this study did not agree with this model, because for the three cases where both the WT and contralateral NR were examined for *CTNNB1*, two showed both the NR and WT were positive for *CTNNB1* mutation while the last case was uninformative.

In a separate study where *CTNNB1* mutations were specifically studied in a patient with germline *WT1* mutation and bilateral WTs, both tumours had a second *WT1* hit of loss of heterozygosity (LOH), while the right tumour had delta45S *CTNNB1* mutation and the left side had S45P in all cell types and a T41A *CTNNB1* mutation specific to a separately microdissected stromal component (Ref. 32). The surrounding kidney was shown to be absent for *CTNNB1* mutation or LOH. These data support *CTNNB1* mutation being a later event in WT tumourigenesis, which is further supported by the fact that new bilateral WTs subsequently developed with novel *CTNNB1* mutations (S45C on the right; S45F on the left) (Ref. 32). A separate study that showed three of five tumours within one patient had different *CTNNB1* mutations (delta45, S45C and S45P) (Ref. 33).

Although the evidence for *CTNNB1* mutation being a late event is inconsistent, these studies, and others (Refs 34, 35) clearly demonstrate that *WT1* mutation can follow the 2-hit tumour suppressor model for the development of cancer. However, the somatic genetics can be complex, with *WT1* mutant proteins demonstrating tumour suppressor functions in some cases and oncogenic properties in others. The differing roles for *WT1* are further supported by the difference in clinical phenotype observed in patients with *WT1* loss and *WT1* mutation; a dominant-negative effect is predicted for intragenic *WT1* point mutations because of the more severe genitourinary phenotype observed in patients with DDS in comparison with patients with complete *WT1* deletion (WAGR syndrome).

#### IGF2 and bilateral WT

In healthy normal tissue, the expression of IGF2 (located at 11p15) is controlled by a nearby imprinting control centre, at which the DNA is methylated on the paternal allele and not methylated on the maternal allele. Expression of IGF2 occurs only when the imprinting control centre is methylated, i.e. from the paternal allele. This normal phenomenon, termed ‘genomic imprinting’ is disrupted in WTs. Somatic biallelic expression because of the loss of the silent maternal allele and duplication of the active paternal allele by LOH is observed in 32% and LOI by gain of methylation is observed in 37% WTs, with overall frequency of around 70% (Ref. 25), reviewed elsewhere (Ref. 36). The low frequency of tumours observed in patients with constitutional LOI may be explained by the presence of mosaicism. 11p15 aberration in lymphocyte DNA has been described in 12% of patients with bilateral WTs and 3% of unilateral sporadic WTs without reported syndromes or associated overgrowth (Ref. 2). Furthermore, mosaic LOI has been reported in the kidney in patients without constitutional aberration (Ref. 37). Therefore, the reverse may be true; that patients with ‘germline’ LOI may show LOI in many tissues, but not the kidney, hence the absence of tumour formation.

In addition to the strong association between constitutional LOI at 11p15 and an increased frequency of bilateral WT, bilateral disease was also significantly more frequent in sporadic WTs with somatic LOI by gain of methylation, compared with tumours without (*P* < 0.001) (Ref. 25) and LOI by LOH was shown to occur infrequently in bilateral tumours compared with unilateral (Ref. 38). Therefore, despite a relatively low penetrance level, LOI by gain of methylation at 11p15 is clearly associated with both unilateral and bilateral WTs, indicating a disruption in normal epigenetic control.

#### Recently discovered WT genes

Besides *WT1* and *IGF2*, several other genes or chromosomes have been analysed in bilateral WTs. Whether these are causative for the predisposition or for the individual tumour analysed remains unanswered and addressing the latter requires detailed analysis of multiple tissue samples from one individual, which is not always achieved in the small series or anecdotal series described. One study highlighted a specific case of bilateral WT in which isochromosome 7q was observed only in the left tumour (Ref. 39). Anaplastic histology, associated with *TP53* mutation, is also frequently discordant between bilateral tumours and hence is believed to be a later event in tumourigenesis (Ref. 40). An example is the longitudinal analysis of a patient with bilateral disease, where *TP53* mutation was not initially detected at diagnosis in biopsies of either side but was found 5 months later in the left kidney tumour (p173V > L), with a different mutation (p195I > T) being found at subsequent relapse 56 months later in the contralateral kidney, where a residual right-sided NR had transformed to WT (Ref. 41). A more recent study analysed *MYCN* and *TP53* status in a pair of bilateral WTs and identified *TP53* mutation and *MYCN* copy number gain in the left tumour with wild-type *TP53* and activating *MYCN* mutation in the right tumour (Ref. 42). When the right kidney suffered a later recurrence, a different *TP53* mutation and wild-type *MYCN* were found, suggesting this was a new tumour rather than relapse of the original. As the relapse was *MYCN* wild-type, gain of *MYCN* was clearly not required for tumour formation; however, it is interesting that both tumours contained activated *MYCN*, albeit by alternative mechanisms. These findings suggest that although *MYCN* gain and *TP53* loss are late events in Wilms tumourigenesis, both molecular aberrations can promote tumourigenesis.

Evidence from mouse models also highlights genetic events that may lead to bilateral disease including the combination of *CTNNB1* mutation with *KRAS* activation in which mice developed bilateral WT-like renal epithelial tumours that were metastatic and multifocal (Ref. 43). However, *KRAS* has not been identified as a human WT-associated gene. On the other hand, *Lin28a* overexpression, led to mainly bilateral tumours (4/5 tumours observed in 50 mice) when it was serendipitously overexpressed from ‘leaky’ expression in a primordial germ cell lineage mouse model experiment (Ref. 44). A further mouse model inducing spatial and temporal control of Lin28a expression in mouse yielded 15 tumours in 15 mice; however, the frequency of bilateral lesions was not discussed. Lin28a human homologue *LIN28B*, was also shown to be overexpressed in the blastemal component of human WT (Ref. 44). LIN28 overexpression is associated with degradation of Let-7 miRNAs, and as Perlman syndrome is associated with *DIS3L2* mutation, the nuclease that degrades poly-uridylated let-7 miRNAs (Ref. 45), and also shows high rates of bilateral WT, this indicates that the miRNA processing pathway may be particularly penetrant for generating bilateral WTs.

More recently, additional genes were found to be mutated in WT, including genes involved in early renal development (*SIX1, SIX2* and *SALL2*) as well as genes involved in the miRNA processing pathway (*DIS3L2, DGCR8, DICER1, DROSHA, XPO5* and *TARBP2*) (Refs 4, 5, 46, 47). It is currently unclear whether there is a link between these novel gene mutations and bilateral disease however mutations in several (*DICER1, DROSHA, DGCR8, XPO5* and *DIS3L2*) have been observed in the germline (Refs 4, 5, 47, 48).

Finally, a very recent article showed intra tumour genetic heterogeneity in WT, bilateral WT appearing genetically distinct and probably arising independently one side from the other (Ref. 49). Such variable heterogeneity will probably become predominant in the near future research to better understand the real genetic landscape of syndromic and nonsyndromic bilateral WT. It may have major implications in the clinical decision-making process to more accurately adapt and personalise treatment strategies for each individual cases.

### Molecular features of NRs and its clinical consequences

By contrast to WT, genetic and molecular studies of NR are scarce because of the difficulty of distinguishing NR from WT and specifically extracting suitable DNA from small microscopic lesions (Ref. 50). *WT1* mutation was identified in the NR of 2/19 patients with WT (Ref. 51) and loss of 11p13 and 11p15 heterozygosity were found in the ILNR of 2/12 patients (Ref. 52). PLNRs also appeared to be associated with IGF2 overexpression and WT in 42 patients but these PLNRs displayed various genomic profiles suggesting that not all PLNR necessarily underwent malignant transformation (Ref. 53). Epigenetic research has recently shown intermediate levels of DNA methylation in NR compared with WT, these methylated regions becoming further methylated with the development of an associated WT (Refs 54, 56). To our knowledge, no molecular analysis of bilateral NRs has been reported so far. In the largest study of patients with diffuse nephroblastomatosis visible on imaging, no molecular analyses were described (Ref. 9).

Thus, differentiating WT from its associated and presumed precursor NR remains challenging on a molecular basis. It would be of great value clinically if imaging features could also contribute to the assessment of this distinction, to predict histological risk group and hence aid with surgical planning of NSS.

### Clinical features

Despite the lack of controlled studies, reports from recently published cooperative, national groups or single institutional series from developing countries with at least 15 patients provide useful data allowing identification of some key features specific to bilateral WT (Refs 20, 56, 57, 58, 59, 60, 61, 62, 63, 64, 65). Clinical characteristics are detailed in Table 1. Age of onset of bilateral WT varied from 15 months to 3.6 years, the lowest being the Japanese series that also presented with the highest rate of associated anomalies, while the highest age of onset was observed in patients from Cape Town that had no associated anomalies (Refs 61, 62). We could argue that better screening of patients followed by paediatricians for other anomalies allows an earlier detection of an abdominal mass. A total of 120 (22%) patients among the 545 listed had associated syndromes or clinically relevant anomalies, the commonest being isolated genito-urinary anomalies (35%), i.e. hypospadias or undescended testis, that were not associated with an already described syndrome. The second most frequent anomaly was isolated hemi-hypertrophy observed in 22 (18.3%) patients. The three syndromes associated with different 11p abnormalities (WAGR, DDS and BWS) were equally represented, ranging from 2 to 3% of the whole cohort and from 9.1 to 14.1% of the patients with clinical anomalies. Conversely, bilateral WT has been reported in 17.3% of BWS patients (Ref. 20), which was three times higher than in the whole WT population, but similar to the rate reported in DDS and WAGR (Refs 18, 19, 20). Hemihypertrophy and nephromegaly have been reported as major risk factors of developing a WT, nephromegaly being particularly linked with bilateral cases (Ref. 66). Molecular features of an unselected series of bilateral WT have only been reported by the Japanese national WT group, who found a high proportion (68%) with *WT1* mutation (Ref. 62). It should be noted however that the Japanese population has a much lower proportion of WT associated LOI at 11p15 than is found in populations of largely Caucasian descent (Ref. 67). No further data on the clinical, radiological, pathological and treatment differences between patients with or without syndromic patterns were displayed in these national series.

**Table 1.** Clinical features of bilateral WT

	AEIOP (Ref. 59)	SFCE (Ref. 64)	NWTS (Ref. 58)	JPLT (Ref. 62)	GPOH/SIOP9 (Ref. 65)	UKW2 (Ref. 60)	Netherlands (Ref. 56)	Egypt (Ref. 63)	Durban (Ref. 57)	Cape Town (Ref. 61)	Total
Number of patients	93	49	188	31	28	70	25	22	20	19	545
Date of study	1990–2011	1993–2001	1986–1994	1996–2011	1989–1994	1980–1995	1967–2007	1993–2008	2002–2012	1981–2003	1967–2012
Median or mean age	24 m [5–86]	2.3 y [1.7 m-8.4y]	32 m [1–127]	15 m [7–62]	1.9 y	24.4 m [1–102]	1.03 y [0.27–5.35]	3y [1–9]	2.5y [10 m-9y]	3.6 y [0.6–7.9]	
Sex	32 M 61 F	18 M 31 F	74 M 114 F	16 M 15 F		28 M 42 F	7 M 18 F	10 M 12 F	11 M 9 F	7 M 12 F	203 M 314 F
BWS	3	0	10	0		2	2		0	0	17
DD	1	4	0	2 with WT1 mutation		3	3		1	0	14
WAGR	2	3	1	0		5	0		0	0	11
Isolated aniridia	2	1	6	0	At least one with 11p13 deletion	0	0		0	0	7
Isolated HH	5	1	15	0		0	0		1	0	22
Perlman	1	1	0	0		2	0		0	0	4
GU anomalies	0	1	6 hypospadias 11 undescended testis	3 hypospadias 4 undescended testis 1 horseshoe kidney		15	0		1 hypospadias with undescended testis	0	42
Other	1 Prune Belly 1 Mental and growth retardation	0	0	1 familial WT		0	0		0	0	3
WT1 mutation				21 (68%)							21
S or Me	All S	All S	All S	All S	All S	All S	14 S 11 Me	19 S 3 Me		14 S 5 Me	506 S 19 Me
Metastatic	11 (12%)	5 (10%)	16 (8.5%)	2 (6%)	3 (11%)	9 (13%)	2 (8%)	0	4 (20%)	1 (5%)	53 (9.7%)

M, male; F, female; m, months; y, years; BWS, Beckwith–Wiedemann Syndrome; DD, Denis-Drash; WAGR, Wilms, Aniridia, Genito-urinary malformations and mental Retardation; HH, hemi-hypertrophy; GU, genito-urinary; S, synchronous; Me, metachronous.

The only national series reporting on bilateral disease associated with nephroblastomatosis presented 52 patients with hyperplastic perilobar nephroblastomatosis, including three patients with unilateral lesions and 49 with bilateral lesions. Among them, 24 developed a WT in their follow-up; 13 a single WT and 11 developed two or more synchronous or metachronous uni or bilateral WTs. The histology of the nephrectomy showed a higher percentage of anaplastic WT (33% of those who developed a WT, 15% of the whole cohort) (Ref. 9). Distinguishing nephroblastomatosis from WT at diagnosis is one of the most difficult aspects of bilateral disease and has clinical significance as the overall prognosis of having a WT associated with nephroblastomatosis led to worse overall and event free survival compared to having an isolated WT (Ref. 68). The study of multiple nephroblastomatosis cases described here showed that the initial biopsy did not aid with distinction in 63% of cases (Ref. 9). Instead, the most reliable pathologic feature seemed to be the presence of a well-defined fibrous pseudo-capsule separating the lesion from the adjacent normal kidney in WT (Ref. 9).

### Radiological features

Bilateral WTs are usually associated with NRs that are small, microscopic lesions not visible on imaging. However, some cases present with one or more expansile lesions seen on imaging. The smallest lesion detectable by ultrasound is at least 8 and 5 mm by CT scan or MRI (Ref. 69). Distinguishing NR from WT is difficult, the most characteristic feature of NR at diagnosis being their diffuse homogeneity both before and after contrast agent administration. After chemotherapy, MRI has been shown to differentiate the active NR and WT (bright on T2 and STIR sequences) from inactive NR and treated WT (dark on T2-weighted images and STIR sequences). The shape of the lesion may aid distinction because of the more oblong or lenticular shape of NRs; however, they can also be spherical like WT, resulting in less than perfect specificity and sensitivity of MRI and CT in the distinction between WT, NR and nephroblastomatosis (Ref. 70).

**Figure 2.**
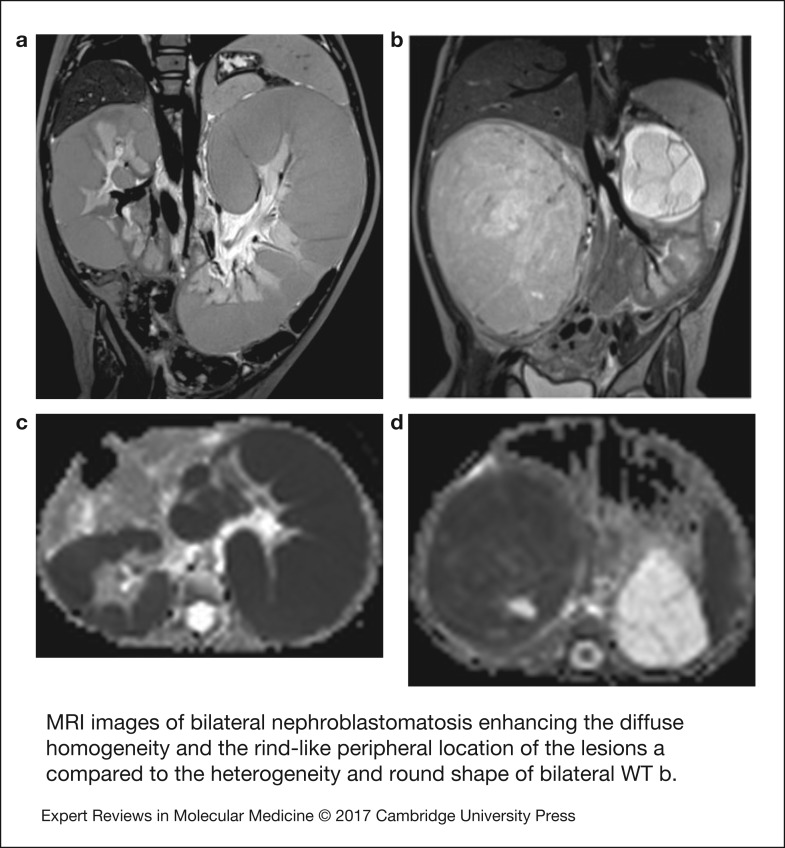
MRI images of bilateral nephroblastomatosis enhancing the diffuse homogeneity and the rind-like peripheral location of the lesions (a) compared with the heterogeneity and round shape of bilateral WT (b). The left kidney seems suitable to a nephron sparing surgery as the mass arises from the superior pole above the left pedicle (b). Corresponding apparent diffusion coefficient (ADC) maps demonstrate low ADC in both kidneys with nephroblastomatosis (c) but different ADC values between the right solid nephroblastoma and the left cystic tumour (d).

Nephroblastomatosis in its diffuse hyperplastic perilobar form is confined to the periphery of the kidneys. Its appearance is usually hypointense to the cortex and isointense to the medulla in MRI nonenhanced T1-weighted images, and hyperintense on T2-weighted images with similar appearance of the cortex. Contrast enhanced MRI or CT make the lesions the most conspicuous (Fig. 2) (Ref. 9).

The recent development of diffusion-weighted MRI in paediatric abdominal tumours (Ref. 71) has shown an inverse relationship between the cellularity of extra cranial tumours and the ADC of these tumours (Ref. 72). The use of ADC measurements to enable differentiation between benign and malignant tumours shows controversial results, potentially explained by a difference in the drawing of the region of interest that should not include any necrotic or cystic area as these areas render ineffective ADC measurement (Refs 71, 72). So far, in WTs, diffusion-weighted MRI has been able to stratify WT histological subtypes with significantly lower values observed in high-risk blastemal-type WTs compared with intermediate risk stromal, regressive and mixed-type. No significant difference in ADC was found between blastemal-type WTs and intermediate risk epithelial-type (Ref. 13). This may be particularly important for identifying the proportion of blastema that has responded to chemotherapy, and the proportion of residual chemotherapy-resistant blastema, as mentioned in the introduction. Ongoing studies are assessing the prognostic significance of these measurements.

### Treatment for bilateral disease

The preoperative chemotherapy regimen favoured when primary surgery was not performed was a course of Vincristine and Actinomycin D, with or without Doxorubicin for a mean duration of about 3 months before the first surgery (Table 2). The timing of performing Nephron NSS or radical nephrectomy reached a consensus on the need to operate before the 12th week of preoperative chemotherapy, first because of the risk of anaplastic transformation (Ref. 73), then because continuing chemotherapy longer will not facilitate conservative resection (Ref. 65) and because nonresponding tumours on radiological assessment may be differentiated tumours (like stromal type) that will not shrink more under further chemotherapy. NSS was performed in 344/517 (66%) patients, combining radical nephrectomy on one side and NSS on the other side (n = 192), bilateral NSS (n = 127), unilateral NSS and biopsy on the other side (n = 11) or unilateral NSS alone (n = 14) (Table 3). Twenty-two additional NSS were performed by the Durban surgical team but with no detail on the side of the surgery (Ref. 57). For central tumours involving the renal hilus, a longitudinal partial nephrectomy was reported in five bilateral WT patients, three of them carrying a *WT1* mutation, with good oncological and outcome results (Ref. 74).

**Table 2.** Treatment modalities and outcome of bilateral WT patients

	AEIOP (Ref. 59)	SFCE (Ref. 64)	NWTS (Ref. 58)	JPLT (Ref. 62)	GPOH/SIOP9 (Ref. 65)	UKW2 (Ref. 60)	Netherlands (Ref. 56)	Egypt (Ref. 63)	Durban (Ref. 57)	Cape Town (Ref. 61)
Number of patients/kidneys	93/176	49/94	188/376	31/56	28/	70/114	25/	22/	20/37	19/
Preop CT	43 VA 37 VAD 1 +Ifo + Carbo 6 primary surgery	VA 10 1st line 6 2nd line (add D or E + C) 4 3rd line	83 primary surgery	11 EE4A 13 DD4A 9 modif (upgrade EE to DD, add C + Carbo) 7 primary surgery	28 VA	13 primary surgery	SIOP protocols since 1971	6 primary surgery 10 VA 6 VAD	10 VA 4 VAD 5 VA*3 1 VA + E + Ifo + cisplat	3 VA 11 VAD 1 VADC 4 VADCE
Duration preop CT	12 weeks (1–40)	80 days (47–89)		14.2 weeks (8–24)						
Histology										
Stage 1	25 (27%)	19 (40%)	63 (17%)		15 (54%)			11 (50%)		6 (32%)
Stage 2	26 (28%)	14 (30%)	215 (57%)		2 (7%)			11 (50%)		12 (63%)
Stage 3	28 (30%)	14 (30%)	73 (19%)		11 (39%)			0	6?	1 (5%)
No stage	14 (15%)	2	12 (7%)					0		0
Low risk	With IR	16 (15%)	?		1			With IR		1 (5%)
Intermediate risk	67 (76%)	78 (83%)	78 (29%)		27			17 (77%)		8 (42%)
High risk (Blastema only)	8 (9%)	0	17 (7%)		0	7 (10%)		5 (23%)	9 (47%)	8 (42%)
Diffuse Anaplasia	10 (12%)	2 (2%)	16 (6%)	0	0	With above		With above	With above	2 (11%)
NR		37 Kidneys (39%)	17 Kidneys (5.5%)			21 (30%)				18 (95%)
Postop CT		4 no postop CT (no response to preop CT) 18 VA 22 VAD 4 VA + other drugs	EE4A DD4A 2^nd^ line in 87 (46%)		Stage 2 regimen	59 VAD 5 VA + C 5 VA 1 V		10 VA 7 VAD 4 VAD + Ifo + Eto 1 died at preop phase		
Radiotherapy	20 (22%)	11 (22%)	64 (34%)	4 (13%)		21 (30%)	5 (20%)	0	13 (65%)	2 (11%)
Relapse	27 (29%)	7 (14%)	54 (28%)	(13%)	(18%)	16 (23%)		1 (4.5%)	2 (10%)	6 (32%)
OS/EFS	80%/66.5% (4y)	89%/83% (5y)	84%/70% (8y)	93%/85% (5y)	85%/80%	69% (6y)	75.6% (20y)	61% (3y)	85% (2y)	51.6%/29.2% (5y) for S 80%/80% (5y) for Me
ESRD	At least 1 (1%)	7 (14%)	23 (12%)	4 (13%)		5 (7%)	8 (32%)	1 (5%)	2 (10%)	2 (11%)

CT, chemotherapy; V, vincristine; A, Actinomycin D; D, doxorubicin; C, cyclophosphamide; E, etoposide; Ifo, ifosfamide; carbo, carboplatin; cisplat, cisplatine; ESRD, end-stage renal disease; NR, nephrogenic rests; S, synchronous; Me, metachronous.

**Table 3.** Surgical management of BWT patients

	AEIOP (Ref. 59)	SFCE (Ref. 64)	NWTS (Ref. 58)	JPLT (Ref. 62)	GPOH/SIOP9 (Ref. 65)	UKW2 (Ref. 60)	Netherlands (Ref. 56)	Egypt (Ref. 63)	Durban (Ref. 57)	Cape Town (Ref. 61)	Total
Number of patients/kidneys	93/176	49/94	188/376	31/56	28/	70/114	25/	22/	20/37	19/	545/>853
Type of surgery					NA						
RN + NSS	31 (33%)	29 (59%)	53 (28%)	15 (48%)		32 (46%)	14 (56%)	13 (59%)		5 (26%)	192
RN + Biopsy	12 (13%)	0	51 (27%)	0		0	0	3 (14%)		0	66
RN + Nothing	With above	0	6 (3%)	0		10 (14%)	0	0		2 (11%)	18
Bilat NSS	35 (38%)	19 (39%)	35 (19%)	10 (32%)		10 (14%)	8 (32%)	3 (14%)		7 (37%)	127
Unil NSS + Biopsy	0	0	10 (5%)	0		0	0	1 (4.5%)		0	11
NSS + nothing	5 (5%)	0	3 (1%)	0		5 (7%)	0	0		1 (5%)	14
Bilat RN	1 (1%)	1 (2%)	6 (3%)	3 (10%)		1 (2%)	3 (12%)	1 (4.5%)		0	16
No surgery	3 (3%)	0	0	0		9 (13%)	0	1 (4.5%)		1 (5%)	14
Other procedures	1 (1%) bilat biopsy 5 unknown	0	19 (10%) bilat biopsy 5 (3%) unilat biopsy	3 (10%) unknown		1 (2%) bilat biopsy 3 (4%) bilat RN + bench surgery + autotransplantation of 1 kidney	0	0	15 RN 22 NSS	3 (16%) bilat biopsy	40 others 15 RN 22 NSS
Surgical complications	1 chylous ascites 1 acute cerebral ischaemia 1 urinary fistula 1 splenic injury 1 bowel obstruction	NA	14 bowel obstruction 1 extensive haemorrhage 1 vascular injury 2 visceral injuries 2 urine leaks 1 urinary obstruction	NA	NA	3 haematuria 1 NSS converted to RN 2 bowel obstruction 1 intestinal perforation 1 diaphragmatic rupture 1 incisional hernia	NA	1 GI bleeding	2 bowel obstruction	NA	40

RN, radical nephrectomy; NSS, nephron sparing surgery; GI, gastro-intestinal; NA, not assessed.

The quality of resection could be evaluated by the number of surgical complications and the number of stage III. Surgical complications occurred in 40/517 (7.7%) patients leading to death in two Italian patients (one chylous ascites and one acute cerebral ischaemia) (Ref. 59) (Table 3). These surgical fatal complications led the Italian group to advocate for a more centralised management of bilateral WT, also noticing that the highest rate of conservative procedures arose from a single expert institution (Ref. 59).

The final pathological analysis showed about 30% WTs were stage III in major series (Refs 58, 59, 64, 65) but without distinguishing radical nephrectomy from NSS. Reasons for stage III were not detailed, but one could argue positive margins as well as omission of lymph nodes sampling that seems more frequent in bilateral WT operated on by NSS (Ref. 75). Despite this high rate of stage III, and the fact that not all stage III patients received radiotherapy, administered at the discretion of the local physician (Refs 59, 64), the event-free and overall survival ranged from 61%/66.5% to 93%/85%, respectively (Table 2). In a single retrospective review of bilateral WT cases, the local recurrence rates after NSS did not prove to be linked to the margin status despite the small number of cases (Ref. 76). However, all patients with positive margins were irradiated. The case for radiotherapy in stage III patients is still a matter of debate as in the French and Italian series, 35 and 43% of stage III patients respectively did not receive radiotherapy and were all alive at last follow-up (Ref. 64).

Unfavourable histology (blastemal type and diffuse anaplasia) ranged from 2% (Ref. 64) to 53% (Ref. 61) (median 21%) and correlated with outcome (Table 2). Like for unilateral WT, histology remains a major risk factor for outcome of bilateral WT, even with adapted postoperative chemotherapy. A real difference in overall survival was also noticed between synchronous and metachronous disease however only one study separated the samples and sample size was small (Table 2) (Ref. 61). Among the studies involving only synchronous disease, the relapse rate ranged from 13 to 29% (Table 2), with around half being only local relapse of whom half were treated by repeat NSS (Refs 58, 77). No details were given on the survival or recurrence rate depending on the presence or absence of associated anomalies or syndromes whether the bilateral disease was synchronous or metachronous.

### End Stage Renal Disease (ESRD) after bilateral WT

The major concern for bilateral WT patients after complete remission of the disease is the evolution of their renal function at long-term follow-up. ESRD was estimated at 0.6% of unilateral nonsyndromic WT but increased to 6.7% for patients with genito-urinary anomalies, 36% for patients with WAGR and 74% for DDS patients (Ref. 78). In cases of bilateral WT, ESRD was 11.5% at a mean of 11.5 years follow-up for nonsyndromic patients, 25% for patients with genito-urinary anomalies, 90% for patients with WAGR and 50% for DDS patients (Ref. 78). Hypertension is another concerning risk at long-term follow-up and has been estimated in a recent analysis of GPOH patients at 66.7% of patients undergoing total nephrectomy on one side versus 20% for patients undergoing bilateral NSS. In a recent single institution review of their bilateral WT operated on by NSS in 92.9% of cases, the authors showed a treated hypertension rate of 30.6% of the 36 living patients at a median follow-up of 3.7 years (Ref. 79). An additional seven patients presented nontreated persistent systolic or diastolic blood pressure readings between the 90th and 95th percentile for their age group increasing the rate of hypertension in the cohort to 50%. The renal function assessed by Schwartz formula showed 36.1% of patients having an estimated glomerular filtration rate of less than 90 ml/min/1.73 m^2^ but none had <60 (Ref. 80).

## Conclusions

Advances in understanding the molecular basis of WT hold much promise for improving the management of the rare but challenging scenario of bilateral disease. Surgical treatment strives to preserve renal function through NSS without compromising complete tumour excision. This is generally facilitated by pre-operative chemotherapy, which brings additional information from assessment of histological response.

Interpretation of the completeness of tumour excision may be confounded by the difficulties in distinguishing NR from fully malignant WT. Here, epigenetic changes may add to current knowledge about the key genetic drivers (*WT1* and *IGF2* disruption) early in renal development and those occurring as later events (*MYCN, TP53* and *CTNNB1* mutation). Recent research has highlighted new pathways associated with WT formation, including mutation of new genes involved in renal development and the miRNA processing pathway. The contribution of mutation in these genes to bilateral disease and, separately, to risk of renal failure, requires further assessment by epidemiological studies in combination with molecular analysis. It is likely that these questions will be answered in a relatively short time scale because of large-scale collaborations and ever decreasing costs of molecular analysis.

There remains a need for noninterventional methods to predict histological subtype so that decisions about intensification of pre-operative chemotherapy and timing of surgery can be planned to maximise the possibility of NSS. Recent advances in MRI diffusion measurements and in detecting circulating tumour DNA may aid in assessment here (Ref. 55). Understanding the full genetic spectrum of bilateral WT is important for treatment planning and follow up to optimise the overall survival of these children, many of whom are expected to have constitutional mutations in WT predisposition genes. These may contribute to their risk of further tumours and of end stage renal failure as well as increased tumour risk in their offspring. Optimum management of bilateral WT requires an experienced multi-disciplinary team with input from the point of diagnosis of all the above specialist areas to achieve the best outcome for each patient.
